# The Na^+^ and K^+^ transport system of sperm (ATP1A4) is essential for male fertility and an attractive target for male contraception^†^

**DOI:** 10.1093/biolre/ioaa093

**Published:** 2020-06-16

**Authors:** Shameem Sultana Syeda, Gladis Sánchez, Jeffrey P McDermott, Kwon Ho Hong, Gustavo Blanco, Gunda I Georg

**Affiliations:** 1 Department of Medicinal Chemistry, College of Pharmacy, Institute for Therapeutics Discovery and Development, University of Minnesota, Minneapolis, MN 55414, USA; 2 Department of Molecular and Integrative Physiology, University of Kansas Medical Center, Kansas City, KS 66160, USA

**Keywords:** ouabain, sperm motility, sperm hypermotility, sperm capacitation, male fertility

## Abstract

One of the mechanisms that cells have developed to fulfil their specialized tasks is to express different molecular variants of a particular protein that has unique functional properties. Na,K-ATPase (NKA), the ion transport mechanism that maintains the transmembrane Na^+^ and K^+^ concentrations across the plasma membrane of cells, is one of such protein systems that shows high molecular and functional heterogeneity. Four different isoforms of the NKA catalytic subunit are expressed in mammalian cells (NKAα1, NKAα2, NKAα3, and NKAα4). NKAα4 (ATP1A4) is the isoform with the most restricted pattern of expression, being solely produced in male germ cells of the testis. NKAα4 is abundant in spermatozoa, where it is required for sperm motility and hyperactivation. This review discusses the expression, functional properties, mechanism of action of NKAα4 in sperm physiology, and its role in male fertility. In addition, we describe the use of NKAα4 as a target for male contraception and a potential approach to pharmacologically block its ion transport function to interfere with male fertility.

## Teaser

This paper reviews the role and mechanisms of action of Na,K-ATPase α4 and its potential use of as a target for male contraception.

## Introduction

**Figure 1:**
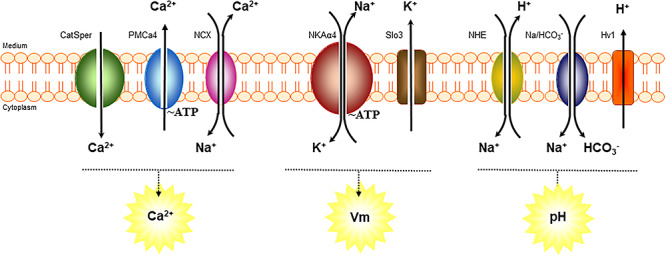
Scheme of key ion transport systems in sperm physiology and relationship of NKAα4 with several of them. NKAα4 actively contributes to maintain the transmembrane Na^+^ and K^+^ gradient and cell membrane potential. PMCA4, plasma membrane calcium ATPase, and NCX, sodium/calcium exchanger, are involved in regulating calcium levels in sperm. SLO3, a K channel that affects cell potassium and membrane potential. NHE, sodium/hydrogen exchanger; Na/HCO_3_^−^, sodium/bicarbonate co-transporter; and Hv1, proton channel, are important for increasing sperm pH.

The rapidly growing world population and high rate of unintended pregnancies make contraception a priority for any public health program. While several contraceptive methods are currently available for women, contraceptive choices for men are limited, and an effective and fully reversible male contraceptive agent is still unavailable [1–4]. Developing a male contraceptive that will meet those characteristics and have the desired selectivity and safety profile represents a difficult task. The strategies for male contraception include pharmacologic as well as barrier-based products that prevent the sperm from reaching the egg. Among the compounds under study are hormonal agents and nonhormonal drugs that target the testis male germ cells or the sperm, either through application to the male or the female [4–6]. The discovery of proteins that are specific to male germ cells of the testis, are absent in somatic cells, and are required for fertility of the male gamete provides an appealing opportunity to achieve reversible male contraception. Sperm function is highly dependent on the exchange of ions between the cells and the medium in which they are immersed. This is under the control of a series of ion channels, exchangers, and active transport systems that operate at the plasma membrane of the cells. These transport systems play a crucial role in triggering events that are key to sperm fertilizing capacity, including capacitation, hyperactivation, and acrosomal reaction, among others. Several of these ion transporters only exist in the male gamete and are structural variants or isoforms, different from the corresponding proteins of somatic cells [7–12]. Some key ion transporters of the sperm plasma membrane and their main function are shown in Figure 1. We are interested in the testis-specific isoform of the NKA and its potential use as a nonhormonal target for male contraception. This manuscript reviews the current status of research in this area.

### Ethics statement

All experimental protocols involving animals in this work were approved by the University of Kansas Medical Center Institutional Animal Care and Use Committee. All authors have participated in the conception, performance and interpretation of the research.

## The Na^+^ and K^+^ transporter, NKA

Compared to their surroundings, animal cells have low Na^+^ and high K^+^ concentrations, which are maintained by the activity of the membrane-bound NKA or Na^+^ pump. This is a plasma membrane embedded enzyme that utilizes the energy from the hydrolysis of ATP to catalyze the movement of intracellular Na^+^ in exchange for K^+^ in a 3 Na^+^: 2 K^+^ fashion [13, 14]. NKA is a member of the P-type class of ATPases, a family of primary transporters that are characterized by forming a transient phosphorylated intermediate from ATP during their functional cycle. The alternating phosphorylated and dephosphorylated states of NKA lead to conformational changes, which are associated with the transmembrane movement of Na^+^ and K^+^ [15]. The asymmetric transmembrane distribution of ions established by NKA participates in the maintenance of cell plasma membrane potential at rest and fuels the Na^+^-coupled transport of many solutes and water across the cell plasma membrane [16–18].

Regarding its structure, NKA is a heterodimer complex, composed of α and β subunits [19–21]. The α polypeptide, considered the catalytic subunit of the enzyme, contains the binding sites for ATP, Na^+^, K^+^, and the NKA inhibitor ouabain. It is a 110–112 kDa protein with 10 transmembrane-spanning domains, with cytoplasmic N- and C-termini, a large intracellular region, and five small extracellular loops [22]. The β polypeptide is a 40–60 kDa single membrane-spanning protein, with most of its mass facing the extracellular medium, where it is heavily glycosylated [19]. The β polypeptide does not directly participate in the transport of ions and the enzymatic activity of the enzyme; however, it plays an important role in the folding, stability, and targeting of the α subunit to the plasma membrane [23, 24].

The heterogeneity in response to the NKA inhibitor ouabain in different tissues provided the first indication that functionally distinct forms of the enzyme existed. Later, evidence for the molecular diversity of the NKA was obtained from the differential migration of the α subunit from different tissues in SDS–polyacrylamide gels (reviewed in [25–29]). With the advent of molecular biological tools, a family of genes encoding for not only different NKA α (NKAα1, NKAα2, NKAα3) but also various NKA β (NKAβ1, NKAβ2 and NKAβ3) polypeptides was discovered in mammals [25, 30–33]. More recently, an additional α polypeptide, NKAα4 (ATP1A4), was identified [34, 35]. The various NKA α subunits are characterized by a high degree of primary structural homology. The β subunits share a lower degree of amino acid identity; however, they exhibit differences in the number and composition of the carbohydrates that form their sugar chains [25, 30].

NKA α and β subunits are expressed in different combinations and in a cell type specific and developmentally regulated manner. While NKAα1 and NKAβ1 are widely present in all cells, NKAα2, NKAα3, NKAβ2, and NKAβ3 exhibit a particular tissue distribution, and NKAα4 is confined to the testis male germ cells. The tissue distribution for the various NKA polypeptides in the rat is shown in Table 1, reviewed in [25]. The α isoform is responsible for most of the functional characteristics that differentiate the NKA isozymes, with the β subunit having only modest effect on the affinity of the NKA for Na^+^ [36–40]. Different NKA αβ dimers result in NKA isozymes that have different functional properties. The recent use of genetic approaches and transgenic technology has allowed a better understanding of the biological role of Na,K-ATPase isoforms in the context of the whole animal [41–46].

**Table 1:** Tissue distribution for the various NKA and 13 isoforms in the rat.

Tissue type	NKA isoforms						
	NKAa1	NKAa2	NKAa3	NKAa4	NKAJ31	NKAJ32	NKAJ33
Testis	X			X	X		X
Sperm	X			X	X		X
Kidney	X				X		
Brain	X	X	X		X	X	X
Heart	X	X	X		X	X	
Lung	X	X			X		X
Liver	X				X		X
Spleen	X				X		X
Intestine	X				X		
Skeletal muscle	X	X			X	X	
Adipose tissue	X	X			X		
Bone	X				X	X	
Peripheral nerve	X	X	X		X	X	
Prostate	X				X	X	
Uterus	X	X	X		X	X	X

Data reviewed in [25].

## The male germ cell-specific isoform of NKA, NKAα4

Early reports have shown that, similar to somatic cells, spermatozoa also maintain transmembrane gradients for Na^+^ and K^+^ [47]. Later, a Na^+^-, K^+^-, and Mg^2+^-dependent ATPase activity was detected in flagellar fractions of boar epididymal spermatozoa [48], and after, this observation was extended to other species [49–52]. Additional studies showed [^3^H] ouabain was able to bind to bull sperm [53]. Moreover, ouabain was found to affect the transmembrane Na^+^ and K^+^ gradients, depolarize the plasma membrane, and reduce flagellar motility of bull sperm [54]. In some species, ouabain also inhibited the acrosomal reaction, a process for fertilization of the egg [55, 56]. Altogether, these results supported the presence of a functional NKA system in the male gamete.

Following these original studies, it was discovered that sperm contained more than one molecular form of the NKA. Early experiments using restriction mapping, Southern blot hybridization, and sequencing of a leukocyte human genomic library allowed the identification of DNA fragments corresponding to partial sequences of a previously unknown P-type ATPase α isoform [35]. This form, originally named αD, resembled an α subunit of NKA, but shared a lower nucleotide identity (between 66 and 76%) with the other Na,K-ATPase α isoforms. It was unclear if this novel partial DNA encoded a functional catalytic form of the NKA or if it corresponded to another closely related cation ATPase. Moreover, the possibility existed that the DNA sequences uncovered represented just a pseudogene. The isolation of the full DNA for the new NKA α isoform and the deduced amino acid primary structure showed that the αD clone was structurally related to NKA. The isoform was then named NKAα4, following the nomenclature used for the other α isoforms; however, its functional validation as a NKA was still unknown [34]. The full sequence of NKAα4 was first reported from rat testis and was shown to correspond to a protein of 1028 amino acid that shared the lowest degree of identity with the other isoforms, with only 78, 78, and 76% amino acid identity with the rat NKAα1, NKAα2, and NKAα3 isoforms, respectively. In contrast, the homology of NKAα4 across species is ~77% [34, 57]. Today, the sequence of NKAα4 is available for a series of species, including human, chimpanzee, gorilla, orangutan, monkey, macaque, bull, buffalo, horse, pig, goat, sheep, dog, cat, rabbit, rat, mouse, gerbil, guinea pig, deer, bear, camel, ferret, lemur, marmot, chinchilla, beaver, cheetah, and opossum. Chromosomal mapping showed that the mouse NKAα4 gene, Atp1a4, is located on mouse chromosome 1. The proximity of Atp1a4 to the Atp1a2 gene that encodes for NKAα2 [58] suggests that NKAα4 may have originated from the NKAα2 gene [32, 58]. Later, the human NKAα4 gene (ATP1A4) was characterized and mapped to chromosome 1q23, and its exon/intron structure was determined [59].

## Identification of NKAα4 as a functional NKA and characterization of its properties

The new NKA gene discovered still required functional confirmation before it could be ascribed as a catalytically competent subunit of NKA. This information came from studies performed in our laboratory on the recombinant NKAα4 protein from rat exogenously expressed in Sf-9 insect cells using the baculovirus expression system [60]. Thus, co-expression of rat NKAα4 and NKAβ1 resulted in an ouabain-sensitive, Na^+^-, K^+^-, and Mg^2+^-dependent hydrolysis of ATP and an ouabain-sensitive uptake of ^86^Rb in the host cells. Also, NKA α4β1 presented an ATP-sensitive phosphorylation from ATP that was inhibited by ouabain, another typical characteristic for a NKA. Furthermore, NKAα4 activity was inhibited by the generic P-type ATPase inhibitor vanadate, but was unaffected by thapsigargin or Sch-28080, compounds that inhibit the sarcoplasmic reticulum Ca^2+^-ATPase or the gastric H,K-ATPase, respectively. In addition, NKAα4 showed an optimal pH for activity of 7.4 and was inactivated by divalent cations, such as Ca^2+^, Cu^2+^, Fe^2+^, and Zn^2+^, demonstrating that H^+^ or divalent cations are not natural substrates of the enzyme and that NKAα4 displays the properties of a NKA [60]. NKA activity with similar characteristics as those of NKAα4 expressed in the insect cells was subsequently found in rat testis [26, 60].

The ability to produce NKAα4 separate from other Na,K-ATPase isoforms in Sf-9 cells allowed us to characterize the enzymatic properties of this NKA isoform [60]. Compared to the NKAα1, NKAα2, and NKAα3 polypeptides, NKAα4 had a relatively higher apparent affinity for Na^+^, a lower apparent affinity for K^+^ and an intermediate affinity for ATP. The biochemical characteristics of rat NKAα4 were also studied in murine NIH 3 T3 cells stably expressing this protein [61]. Analysis of NKAα4 interaction with Na^+^ and K^+^ in [^3^H] ouabain binding/displacement experiments further proved that NKAα4 exhibited the characteristics of a NKA and not those of another P-type ATPase. Interestingly, through inhibition of NKA activity by ouabain, NKAα4 was shown to exhibited high sensitivity to ouabain, with a calculated IC_50_ in the low nanomolar range [26, 60]. [^3^H] ouabain binding self-competition assays confirmed that NKAα4 had high affinity for ouabain, although the calculated dissociation constant resulted to be slightly higher than those of previous reports, which could depend on differences in the membrane preparations used in each study [26, 60]. The high ouabain affinity of NKAα4 was also reported for the human isoform, by assessing the survival of HeLa cells expressing NKAα4, to increasing concentrations of ouabain [62]. Later, direct measurements of the enzymatic properties of NKAα4 in human sperm have shown that NKAα4 affinities for Na^+^, K^+^, and ouabain are conserved with those of the rat ortholog [63].

## Tissue expression and cell localization of NKAα4

Studies in the rat and mouse have shown that NKAα4 is the NKA isoform with the most restricted pattern of expression, being uniquely present in the testis. However, the testis does not exclusively express NKAα4; the ubiquitous NKAα1 is also present in the male gonad. In contrast, rat testis does not express the NKAα2 and NKAα3 isoforms [26, 34]. In vitro hybridization and immunochemical techniques have shown that NKAα4 is present only in the testis seminiferous tubules, where it is abundant in the luminal side of the tubules [64, 65]. Accordingly, NKAα4 is found in most mature male germ cells and in spermatozoa, but not in Sertoli, Leydig, or undifferentiated male germ cells. Different from NKAα4*,* NKAα1 is expressed in all cells of the testis. These data show that NKAα4 localizes to the testis male germ cells. Analysis of the ouabain inhibition profile of NKA activity has revealed that, in rat sperm, approximately two thirds of the total NKA correspond to NKAα4, the remaining being NKAα1 [66]. The primary expression of NKAα4 in sperm was further supported by the drastic reduction of the protein in mice that are oligospermic due to ablation of Egr4, a the transcription factor that is essential for spermatogenesis [64].

Immunocytochemical studies in rat, mouse, and human sperm have shown that NKAα4 is expressed in the flagellum, being more abundant in the midpiece of the sperm tail. Little or no NKAα4 is found in the sperm head [63, 65, 66]. However, while human NKAα4 was reported by one study to be mainly localized in the sperm flagellar midpiece, in another study it was shown in the principal piece, a fact that may depend on the antibodies used in each study [62, 63]. Different from NKAα4, the NKAα1 polypeptide appeared to be more evenly distributed along the sperm flagellum and was barely detected in the sperm head [66]. This suggests the existence of isoform-specific mechanisms for the targeting and retention of NKAα1 and NKAα4 at particular regions of the sperm plasma membrane. Further evidence for the flagellar distribution of NKAα4 was obtained in transgenic mice overexpressing the rat ATP1A4 tagged at its C-terminal portion with GFP [67]. However, staining for GFP extended beyond the midpiece and into the principal piece of the flagellum, which may depend on changes in NKAα4 targeting to the cell surface due to the addition of GFP to the protein or overloading of the protein delivery mechanisms in the cells as a consequence of NKAα4 overexpression. Different from mouse, rat, and human, bull sperm appears to express NKAα4 mainly in the cell head and the post acrosomal region, depending on the non-capacitated or capacitated state of the cells, respectively [68]. Bovine sperm appears to also be peculiar with respect to the isotype of NKA isoform expressed, since it contains NKAα3 and NKAβ2, which are not found in human, rat, or mouse sperm [69]. Interestingly, at the plasma membrane, NKAα4 has been found expressed both in lipid rafts and non-raft fractions of the sperm plasma membrane [70]. In conclusion, subcellular localization studies indicated a species-specific localization for NKAα4, with a primary flagellar distribution in most species and a predominant head compartmentalization in the bovine. A comparison of the NKA isoform composition, NKAα4 gene homology, expression, cell localization, enzymatic properties, and role in sperm function for the species in which most of the sperm NKA studies have been performed is shown in Table 2.

**Table 2:** Comparison of the characteristics of the NKA and NKAa4 isoform in species in which most of the studies have been performed.

	Human	Rat	Mouse	Bull
NKA isoform composition in sperm	HKAa1, NKAa4, NKAl31, NKAl33	HKAa1, NKAa4, NKAl31, NKAl33	HKAa1, NKAa4, NKAl31, NKAl33	NKAa1, NKAa3, NKAa4, NKAl31, NKAl32, NKAl33
Main localization of NKA isoforms in sperm	Flagellum, midpiece	Flagellum, midpiece	Flagellum, midpiece	Head (NKAa1 equatorial region; NKAa3 in post-equatorial region)
Chromosome location of NKAa4	1q23.2	13q24	1H3	3
NKAa4 homology comparison between species	83% (rat), 83% (mouse), 87% (bull)	83% (human), 94% (mouse), 82% (bull)	83% (human), 94% (rat), 83% (bull)	87% (human), 82% (rat), 83% (mouse)
Enzymatic properties of NKAa4 relative to other NKA isoforms within the same species	High affinity for Na^+^, low affinity for K^+^	High affinity for Na^+^, low affinity for K^+^, intermediate affinity for ATP	High affinity for Na^+^, low affinity for K^+^, intermediate affinity for ATP	Non-determined
Ouabain IC_50_	High sensitivity to ouabain (nM)	High sensitivity to ouabain (nM)	High sensitivity to ouabain (nM)	Not exactly determined
Role in sperm	Motility and hypermotility	Motility and hypermotility	Motility and hypermotility	Motility, capacitation, and acrosomal reaction

## Regulation of NKAα4 expression during spermatogenesis

Immunocytochemical studies on rat seminiferous tubules have shown that expression of NKAα4 is highest in spermatozoa and lower in the undifferentiated male germ cells [65]. Also, NKAα4 is scarce in GC-1 cells, a male germ cell line which does not completely mature in vitro [64]. These findings were the first indication that NKAα4 expression was regulated during spermatogenesis. Northern blot analysis of rat testis RNA showed that NKAα4 is not expressed until 4 weeks of age, reaching peak levels at week 6. In contrast, RNA for the NKAα1 isoform in testis was found to remain relatively constant throughout the life of the animal [65]. Immunocytochemical studies showed that NKAα4 expression starts at 6 weeks of age, with maximal expression levels at 8 and 12 weeks of age. Therefore, NKAα4 protein expression closely followed that of RNA [65]. Overall, these results indicated that NKAα4 expression is regulated during development and coincides with the onset of sexual maturity in the rat.

**Figure 2:**
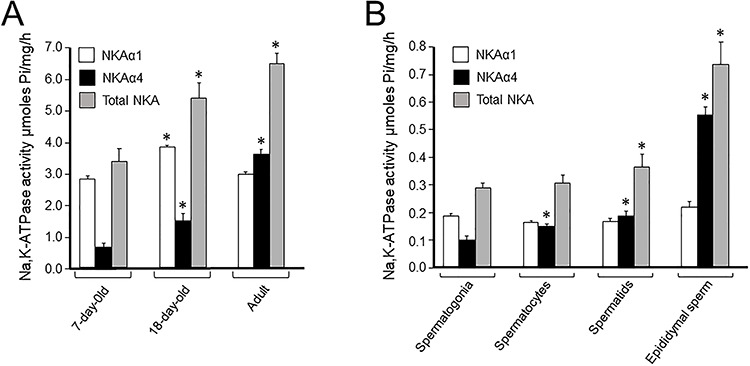
Activity of total NKA and the NKAα4 and NKAα1 isoforms during development. (A) Specific activity measured on rat testis homogenates at 7 and 18 days after birth and in adult animals. Specific activity was measured on (B) NKA activity measured in homogenates from the enriched preparations of different male germ cell types, isolated by unit gravity sedimentation. Total Na,K-ATPase was determined as the hydrolysis of ATP sensitive to 1 mM ouabain. Activity of NKAα4 was measured as the ATP hydrolysis sensitive to 1 μM ouabain, while function of NKAα1 was obtained by the difference between 1 μM and 1 mM ouabain. Values are the mean, and bars represent the SEM of three to five experiments. The asterisks indicate values significantly different from the corresponding samples at day 7 (A) or spermatogonia (B), with *P* 0.05–0.001 [66].

We have found that absolute values of NKA activity on testis homogenates increased approximately two-fold between week 1 of age to adulthood [66]. NKA activity assays for the NKAα1 and NKAα4 isoforms in testis homogenates, distinguished by their differences in ouabain sensitivity, showed that the relative contribution of each α isoform to the total NKA of the gonad varied with age. ATP hydrolysis by NKAα4 increased from 10% of the total NKA of the testis at week 1, to approximately 20% at day 18 after birth, and became almost half of the total NKA in the adult gonad. Instead, the activity of NKAα1 remained relatively constant throughout those timepoints [66] (Figure 2A). In agreement with these results, immunoblot analysis has shown that NKAα4, but not NKAα1, increases during maturation of the male gonad [66]. Thus, NKAα4 levels raised from 1 week after birth, in which spermatogonia predominate in the developing testis, to 18 days of life, in which preleptotene, leptotene, and pachytene spermatocytes are present, and become even higher at adulthood, where cells at all stages of spermatogenesis, including spermatids and spermatozoa, are present [71]. These results showed that NKAα4 expression correlates with sexual maturation of the testis and the onset of sperm formation. A more refined study, performed in highly enriched fractions of different male germ cell types, obtained after testis cell dissociation and unit gravity sedimentation, or counterflow elutriation [66, 72], confirmed the developmental upregulation of NKAα4. In those studies, ouabain inhibition profiles of NKA activity showed that spermatogenesis was accompanied by an approximately two-fold increase in absolute values of total NKA activity and approximately six-fold increase in NKAα4, with minimal changes in NKAα1. NKAα4 activity was low in undifferentiated spermatogonia, augmented in pachytene spermatocytes and round spermatids, and was maximal in spermatozoa (Figure 2B). RT-PCR and immunoblot analysis agreed with the functional assays, showing upregulation of NKAα4 transcription in pachytene spermatocytes, followed by a high protein synthesis later during spermatid development and in spermatozoa [66]. These results have shown that NKAα4 has a postmeiotic pattern of expression, which supported the idea that it had an important role for the differentiated sperm.

To further determine the temporal and spatial pattern of expression of NKAα4, we used the green fluorescent protein (GFP) as a reporter for the activity of the ATP1A4 gene promoter. This approach has confirmed that the ATP1A4 promoter drives the testis-specific expression of GFP and it is responsible for the absence in expression in other tissues. In addition, GFP expression was developmentally regulated, appearing in adult, but not in mouse embryos or sexually immature 7 and 18 day old mice [73]. Immunocytochemistry of whole testis sections identified GFP only in differentiated male germ cells, but not in spermatogonia, Leydig, or Sertoli cells. Further studies in the fluorescently sorted testis cells from those mice, and the use of cell type-specific markers detected NKAα4 in spermatocytes, spermatids, and spermatozoa [73]. Altogether, these studies have provided evidence, beyond that of previous studies that the ATP1A4 promoter drives expression of NKAα4 exclusively at late stages of spermatogenesis of the testis male germ cells. This postmeiotic expression pattern of NKAα4 is shared with that of other genes that play an essential role for sperm function [74].

We explored the transcriptional mechanisms regulating NKAα4 expression of the human *ATP1A4* gene [75]. To achieve this, we analyzed a region of approximately 1 kb upstream the first methionine codon of ATP1A4, which had been predicted as the proximal promoter region of the isoform by *in silico* studies [59]. We identified that this 5′ untranslated region of the ATP1A4 gene exhibits promoter activity in luciferase reporter assays and found the transcription initiation site of the ATP1A4 promoter to an adenosine located 472 bp upstream of the ATP1A4 start codon. Within this region, we observed two consensus sites for the cyclic AMP (cAMP) response element modulator (CREMt), a testis-specific splice variant of the transcription factor CREM [74]. We found that CREMt, in the presence of cAMP, is an important activator of the ATP1A4 promoter and characterized its activity [75]. In the native environment of the testis, CREMt expression is temporally coincident with upregulation of a series of postmeiotic genes [76]. Therefore, the transcriptional regulation of ATP1A4 gene expression by CREMt places ATP1A4 within the cluster of genes that are upregulated after meiosis and serves a key role in sperm function.

## Function of NKAα4 and its relevance to sperm function

**Figure 3:**
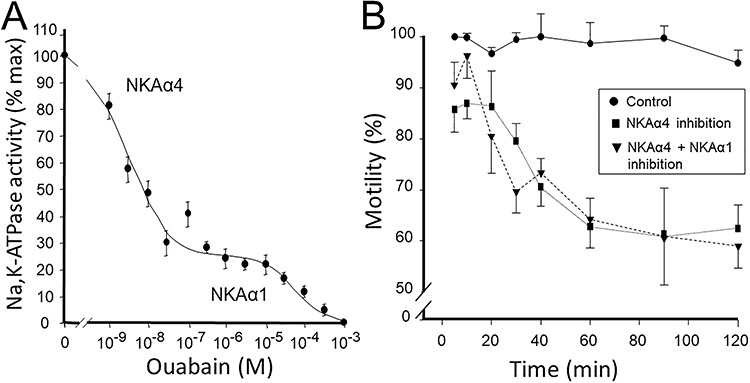
(A) Dose–response curve for the ouabain inhibition of NKA from rat sperm. NKA activity was measured under saturated conditions of Na^+^, K^+^, and Mg^2+^ and at the indicated ouabain concentrations. Curves represent the best fit of the experimental data and indicate the presence of two enzyme components, one highly sensitive (NKAα4) and the other one resistant (NKAα1) to ouabain. Each value is the mean, and error bars represent the SEM of three experiments performed in triplicate. (B) Measurement of sperm total motility using computer-assisted sperm analysis (CASA) on rat epididymal spermatozoa. Cells in Whittens medium were treated for the indicated times with no ouabain (control), 1 nM ouabain (NKAα4 inhibition), or 1 μM ouabain (NKAα4 plus NKAα1 inhibition). Sperm motility was measured under non-capacitating conditions. Values are mean ± SEM [66, 77].

The cell type developmentally regulated expression, and its unique enzyme kinetics suggested that NKAα4 would perform a specific function in sperm. The high difference in ouabain affinity of NKAα4, which is much higher than that of NKAα1 [25], provided the opportunity to selectively inhibit NKAα4 and determine its function, separate from that of NKAα1, the only other NKA α subunit expressed in sperm (Figure 3A). Initial studies in rat were directed to test if ouabain inhibition of NKAα4 affected sperm motility using simple visual determinations of sperm movement [65]. Then, the introduction of computer-assisted sperm analysis (CASA) provided higher resolution for the analysis of NKAα4 action on different parameters of flagellar beat. Inhibition of NKAα4 with 1 μM ouabain to completely block NKAα4 reduced total sperm motility (Figure 3B). These ouabain amounts also interfered with different components of flagellar beat, including progressive motility, straight line, curvilinear and average path velocities, lateral head displacement, beat cross-frequency, and linearity [77]. Higher concentrations of ouabain (1 mM), which also inhibited NKAα1, did not cause additional reduction in sperm motility [65, 77]. These results have revealed the specific role that NKAα4 plays in supporting sperm flagellar beat and its capacity to support multiple parameters of sperm movement.

From these experiments, the idea developed that the ubiquitous NKAα1 contributes to maintain sperm basal Na^+^ and K^+^ transport in sperm and that NKAα4 functions to fulfill sperm-specific roles. Ouabain inhibition of NKAα4 causes depolarization of the sperm plasma membrane [77]. This shows that in sperm, as in other cell types, NKA function is linked to sperm K^+^ channels to maintain the resting, as well as the action membrane potential. An adequate membrane potential is essential for sperm motility, and hyperpolarization of the plasma membrane is key to sperm capacitation. Supporting this is the association that exists between cell membrane depolarization and infertility in asthenozoospermic patients [78]. Therefore, one of the mechanisms by which NKAα4 isoform influences sperm motility is through its key role in maintaining the uneven transmembrane distribution of Na^+^ and K^+^ and the electrical potential across the sperm plasma membrane. Besides its direct role in Na^+^ and K^+^ transport, NKAα4 has high affinity for Na^+^, which makes it best suited to maintain the low intracellular Na^+^ ([Na^+^]*_i_*) levels in sperm. The importance of the transmembrane Na^+^ gradient for the co- and counter transport of other solutes in the cell highlights the role of NKAα4 in sperm function. Thus, NKAα4 secondarily controls proton levels in rat spermatozoa, as shown by the decline in pH of the sperm cytoplasm after selective inhibition of NKAα4 with ouabain [65, 77]. The effects on pH appear to be mediated via the Na^+^/H^+^ exchanger (NHE). This is supported by the finding that (1) several NHE transporters are expressed in sperm, including the somatic cell NHE1 and NHE5 and the sperm-specific NHE (sNHE) [79] and (2) NHE1 and NHE5 are co-localized with NKAα4 [80], and the effect of the ionophores nigericin and monensin, which by inducing H^+^ movement out of the cells, is able to reestablish the inhibition of sperm motility produced by ouabain [80]. Therefore, in rat sperm, NKAα4 prevents the rise in protons that are generated upon active movement of the cells [65].

**Figure 4:**
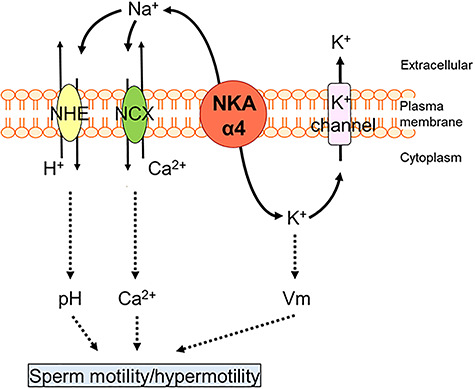
Mechanism of action of NKAα4. The Na^+^ gradient created by NKAα4 is used by secondary transport mechanisms (NHE and NCX) to maintain appropriate sperm cytosolic H^+^ and Ca^2+^ levels. Also, NKAα4, along with K^+^ channels, contributes to sperm membrane potential (Vm). By supporting these important cell parameters, NKAα4 controls sperm function and fertility. Solid lines show direct functional association; dotted lines represent the possibility of indirect effects and additional pathways.

We have also found that NKAα4 is functionally coupled to the regulation of sperm Ca^2+^ and that ouabain inhibition of NKAα4 increases sperm intracellular calcium ([Ca^2+^]*_i_*) [77]. Since our experiments were performed in the absence of extracellular Ca^2+^, the increase in [Ca^2+^]*_i_* is not due to Ca^2+^ internalization from the media, but rather depends on a decrease in Ca^2+^ clearance from the cell cytoplasm, possibly via the Na^+^/Ca^2+^ exchanger (NCX). NCX is expressed at the sperm flagellum, which supports the functional coupling between this ion transporter and the NKAα4 [81, 82]. Due to the key role of [Ca^2+^]*_i_* in sperm motility [81], regulation of this cation may be another mechanism by which NKAα4 sustains sperm motility. A scheme showing the mechanisms by which NKAα4 influences sperm function is shown in Figure 4. Different from rats, ouabain effects on bull sperm did not affect [Ca^2+^]*_i_* and, instead, inhibited progressive but not total sperm motility [56]. These differences may be reflecting dissimilarities in species, in the amounts of ouabain, or in the incubation times used, which were different in the various species. Interestingly, binding of ouabain to bull sperm induced the activation of kinases and the phosphorylation of proteins in tyrosine residues. Moreover, the effect of ouabain in bull sperm was accompanied by an activation of sperm capacitation [83]. These effects are presumably taking place via the capacity of the NKA to function not as an ion transporter, but as a receptor and signal transducer of ouabain effects in cells [56]. Accordingly, both NKAα1 and NKAα4 have been found to associate with raft and non-raft lipid domains of the bull plasma membrane with the epidermal growth factor, the kinase Src, and the extracellular regulated kinase ERK, proteins known to be part of NKA signaling system [68]. Further studies are needed to ascertain the species dissimilarities in the response of sperm to ouabain.

## NKAα4 function during sperm capacitation

Our studies in rat showed that sperm capacitation was accompanied by a time-dependent increase in ion transport and enzymatic activity of NKAα4 [84]. This indicates that NKAα4 activity is stimulated as sperm becomes capacitated. Interference of NKAα4 activity with ouabain blocks the increase in sperm motility and prevents the plasma membrane hyperpolarization and hyperactive pattern of sperm motility that is commonly associated with sperm capacitation. Concomitant with the increase in NKAα4 activity, we found a capacitation-dependent increase in labeling of sperm with the fluorescent indicator BODIPY-ouabain and an increase of NKAα4 at the sperm plasma membrane [84]. Therefore, it appears that NKAα4 is regulated via mechanisms that involve increases in the molecular activity of the ion transporter and an increase in NKAα4 levels at the sperm surface. Since mature spermatozoa are thought to be transcriptionally and translationally silent, the increase in NKAα4 at the plasma membrane could be due to translocation of the protein from intracellular compartments to the sperm surface. Alternatively, in bull sperm, it has been shown that NKAα4 increase can take place by mitochondrial ribosome-associated translation [85]. We are currently performing additional experiments to ascertain the location and molecular mechanisms involved in the subcellular translocation of NKAα4 in mouse and human sperm. In any case, the existence of regulatory mechanisms for NKAα4 highlights the role of this ion transporter in sustaining the changes that sperm undergoes during capacitation and that are important for their fertilizing capacity.

**Figure 5:**
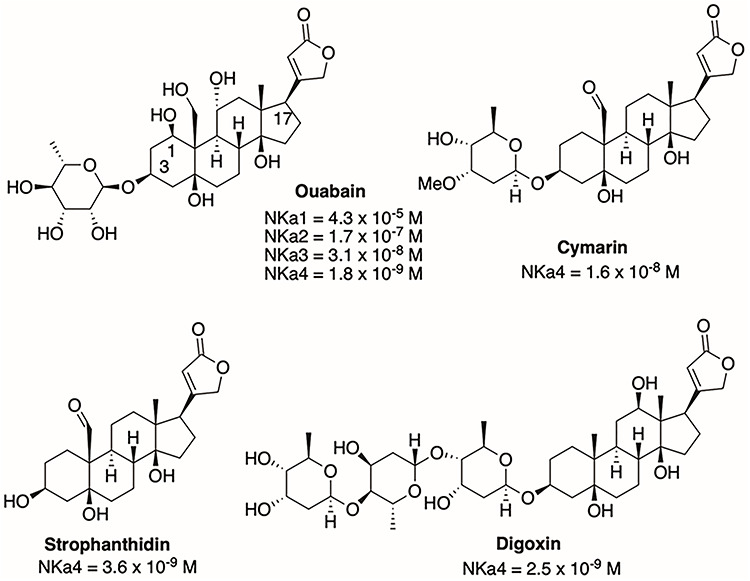
Structures of ouabain, cymarin, digoxin, and strophanthidin and IC_50_ values for inhibition of several NKA isoforms.

## NKAα4 and male reproduction

To directly assess the overall role of NKAα4 in male fertility, we engineered genetically modified mice, in which NKAα4 was either deleted or overexpressed [67, 86]. Knockout mice were obtained by removing exons 5 to 8 of the Atp1a4 gene, which encodes for the ATP binding and phosphorylation sites of the catalytic domain of NKAα4. Homozygous knockout male mice lacked expression of NKAα4 mRNA and protein and showed significantly lower level of total NKA activity. While activity of NKAα1 was still present, the high ouabain affinity activity corresponding to NKAα4, as well as BODIPY-ouabain binding capacity, was lost. NKAα4-null mice were overall phenotypically normal, showing testis with size and morphology indistinguishable from wild-type mice. Also, the NKAα4 knockout mouse presented normal sperm numbers. However, the homozygous male, but not the female mice, was completely infertile. Heterozygous male mice were fertile, suggesting that partial expression of NKAα4 is sufficient to support male fertility [86]. Sperm from the NKAα4 null mice showed severe reduction in all parameters of sperm flagellar beat, lacked the hyperactive pattern of movement, and were incapable of fertilizing oocytes in vitro. Also, Atp1a4 null mice show increased intracellular Na^+^ and Ca^2+^ levels, acidification of the cytosol, and a depolarized plasma membrane [86]. This recapitulates the effects that ouabain inhibition of NKAα4 has on sperm [77]. Other alterations of sperm from NKAα4-null mice consisted in a bend in the sperm flagellum, possibly due to abnormalities related to osmotic imbalance in the cells. The lack of NKAα4 expression in the mice was not accompanied by compensatory upregulation of expression and activity of NKAα1 [86]. Overall, these results agree with the NKAα4 pattern of expression, most in mature germ cells and not in Sertoli or undifferentiated germ cells, and indicate that NKAα4 is not required for spermatogenesis, but it is essential for male fertility.

Further evidence for the role of NKAα4 in sperm function was obtained in transgenic mice overexpressing the rat NKAα4 fused at the C-terminus with green fluorescent protein (GFP), under the protamine-1 promoter [67]. These mice showed an approximately 20% increase in total sperm motility. Mating trials with WT females showed that despite having higher motility, transgenic mice expressing NKAα4 had similar fertility than WT mice, an event that is not surprising, considering that the increase in fertility is limited to the female factor. Therefore, NKAα4 supports sperm flagellar beat, and changes in its expression influence sperm swimming capacity. In agreement with this, a correlation has been found between the expression levels and activity of NKAα4 with male fertility in patients presenting asthenozoospermia [87].

## NKAα4 as a target for male contraception

Due to its key role in male fertility, targeting NKAα4 offers several advantages for the achievement of male contraception: (a) NKAα4 is located on the sperm surface, which facilitates its pharmacological reach; (b) NKAα4 expression in sperm provides selectivity of effect while minimizing global body toxic effects; (c) NKAα4 late appearance in spermatogenesis reduces the possibility of affecting progenitor male germ cells, which allows temporary and reversible inhibition of male fertility [9]; and (d) sperm can be targeted at multiple sites, in the testis, epididymis, the ejaculate via accessory gland secretions, and even in the female genital tract.

The high affinity of NKAα4 for ouabain, which is ~1000–10 000 fold higher than that of the other NKA isoforms, suggests that ouabain is an attractive scaffold for the development of compounds that can target NKAα4 and induce male infertility. Relevant to this is that a correlation has been observed between the presence of higher than normal endogenous ouabain levels in seminal fluid of asthenozoospermic patients consulting for infertility [88]. Ouabain binds to NKA with high specificity; however, at the NKA isoform level, it is only dose selective, and it can eventually inhibit all NKA isoforms. It is clear that the use of ouabain will have negative secondary effects in the body and that compounds with better selectivity for NKAα4 will be required. We exploited the high ouabain affinity of NKAα4 to develop compounds based on the structure of ouabain to specifically target NKAα4 and produce male infertility [89].

Ouabain belongs to a family of compounds known as the cardenolides, which bind to NKA with high specificity. Cardenolides consist of a steroidal nucleus or aglycone; depending on the type of cardenolide, a five-membered unsaturated lactone ring attached at position C17 of the steroid backbone; and a specific sugar moiety, or glycone, attached at position C3 [90, 91]. We first studied different commercially available cardenolides without and with sugar moieties of various lengths (ouabain, strophanthidin, cymarin, and digoxin) (Figure 5) and tested for their capacity to inhibit NKAα4 produced in Sf-9 insect cells using the baculovirus expression system. The results showed that the carbohydrates in cardenolides did not significantly affect their inhibitory capacity or selectivity for NKAα4 [89].

**Figure 6:**
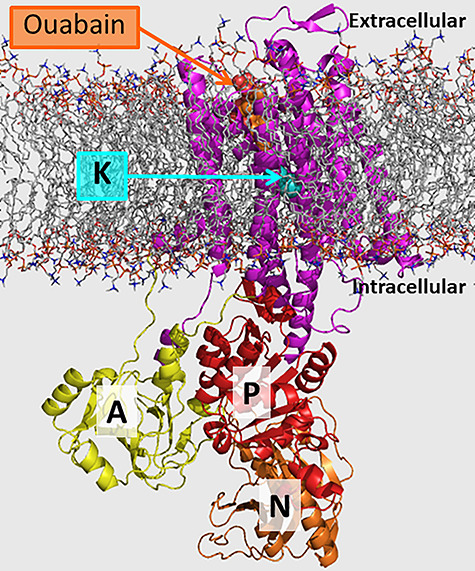
Homology model of rat NKAα4 with ouabain and K^+^ ions. Ouabain (orange) binding pocket is located at the extracellular side of the transmembrane domain (magenta), alkali metal binding site at the transmembrane domain (K, K^+^, and Na^+^ bind to this site), actuator domain (A, yellow), phosphorylation domain (P, red), and nucleotide-binding domain (N, orange) at the intracellular region.

To help guide compound synthesis, we performed an in silico analysis of our compounds, starting by modeling human and rat NKAα4 based on the known crystal structure of the NKAα1 isoform [22, 92]. Overall, the NKAα4 structure is similar to that of NKAα1, presenting the three typical actuator (A), nucleotide-binding (N), and phosphorylation (P) domains of NKA (Figure 6). Our modeling predictions agreed with site-directed mutagenesis studies, which showed that amino acids in the extracellular loops between TM1-TM2, TM3-TM4, TM5-TM6, and TM7-TM8 are important for ouabain binding [93–96]. We found that the ouabain binding pocket of NKAα4 is smaller than that of NKAα1, which may explain the tighter binding and higher ouabain affinity of NKAα4 compared to NKAα1 [89]. With a structural model for NKAα4 available, docking simulations assisted with the analysis of new compound binding capacity to NKAα4 and are a valuable approach to help predict binding of compounds to NKAα4.

**Figure 7:**
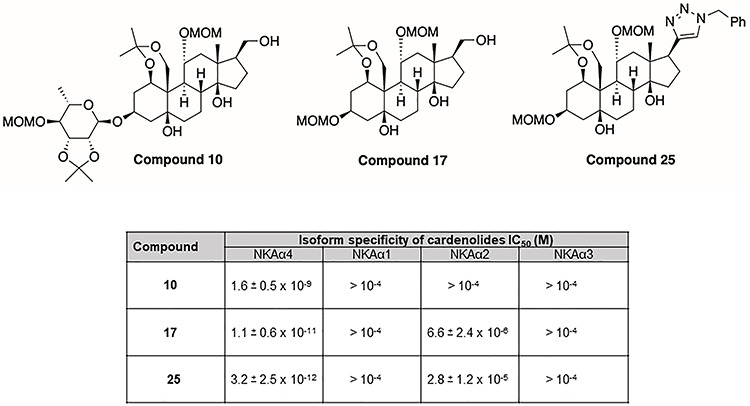
Structure of analogs **10**, **17**, and **25** and their IC_50_ values for inhibition of NKA isoforms. The IC_50_ values were calculated from dose–response curves of inhibition of Na,K-ATPase α1β1, α2β1, α3β1, and α4β1 expressed in Sf-9 insect cells. Values are the mean ± SEM of three experiments [90].

**Figure 8:**
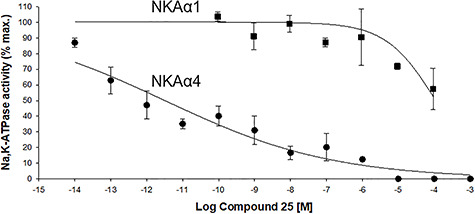
Selectivity of compound 25 for NKAα4 inhibition. Dose–response curves for the inhibition of NKA activity by compound **25** was determined on the NKAα1 and NKAα4 isoforms produced after heterologous expression in insect cells. NKA activity was measured under saturated concentrations of Na^+^, K^+^, and Mg^2+^. The curves represent the best fit of the experimental data, considering a single population of ouabain binding sites. Values are mean ± SEM [90].

We synthesized new compounds in which the aglycone and the lactone ring domains of ouabain were modified. As predicted from the previous studies with the cardenolides shown in Figure 5, ouabain analogs without sugar moiety such as compounds **17** and **25** were highly potent (Figure 7). We also found that protection of the ouabain hydroxyl groups as methoxymethyl ethers or as acetals (at the carbohydrate moiety or between the hydroxyl group at C1 and the C10 hydroxymethyl group, Figure 7) was highly potent, indicating that these hydroxyl groups are amenable to significant structural modifications without loss of potency or even are enhancing potency of the analogs compared to ouabain. The 2-butenolactone group at C17 of cardenolides is important for receptor binding through hydrogen bonding interactions. We therefore designed analogs in which we employed 1,2,3-triazoles and hydroxymethylene-linked triazoles as bioisosteres of the C17 lactone [97]. Other C17 modifications included a nitrile, an oxime, and an aldehyde moiety. Most of the analogs prepared and tested retained significant potency at single- and double-digit nanomolar potencies for NKAα4 inhibition with the exception of those analogs that carried the C17 nitrile group (IC_50_ = 16 μM) or a C17 ethanediol moiety (IC_50_ = 2.1 μM). Two of the most potent analogs, compounds **10** and **17**, feature C17 hydroxymethyl groups, a finding that is worth exploring further. Ouabain analogs **17** and **25** showed subnanomolar NKAα4 activity inhibition and were tested together with nanomolar analog **10** for isoform specificity (Figure 7). All three analogs showed selectivity that was several orders of magnitude higher for NKAα4 inhibition than for the other three isoforms. A comparison of the preferential inhibitory activity of compound **25** for NKAα4 over NKAα1 is shown by the dose inhibition curves of NKA activity performed on the corresponding proteins expressed in insect cells (Figure 8). Testing of the three analogs for inhibition of sperm motility led to the selection of compound **25** for further study.

**Figure 9:**
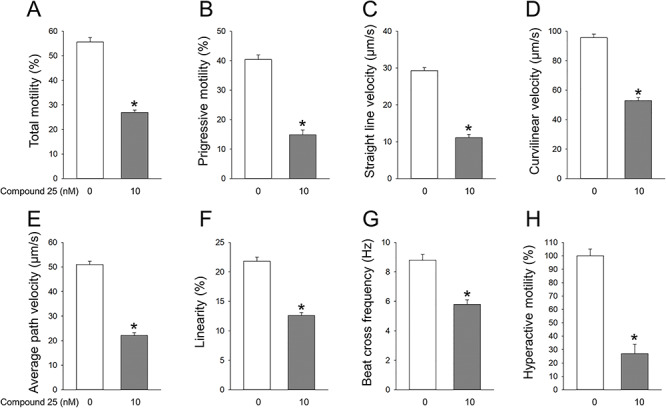
Effect of compound **25** on different parameters of motility of rat epididymal sperm. Sperm was treated in the absence and presence of the indicated concentrations of compound **25**. After 1 h incubation, different parameters of sperm movement were determined with CASA. (A) Total motility, (B) progressive motility, (C) straight line velocity, (D) curvilinear velocity, (E) average path velocity, (F) linearity, (G) beat cross-frequency, and (H) hypermotility measured after sperm incubation in capacitation medium for 1 h.

Compound **25** inhibited total motility and most parameters of motility of rat epididymal sperm, with a maximal reduction achieved at amounts of 10 nM and higher. CASA analysis indicated a decrease in total sperm motility by compound **25** of approximately 60%, with varying inhibition profiles for progressive motility, straight line velocity, curvilinear velocity, average path velocity, linearity, and beat cross-frequency (Figure 9A–G). While the effect of ouabain derivatives on sperm motility is not maximal, the reduction in sperm motility reaches approximately 60%. This approximates to the lower reference limits for normal sperm motility established by the World Health organization (WHO), which define a motility of 40% or less as a predictor of male infertility [98]. A major inhibitory effect of compound **25** was on hyperactive motility, the movement pattern typical of capacitated sperm, where an inhibition of ~75% was reached (Figure 9H). The binding of the compound to sperm appears to be relatively stable, since inhibition of sperm motility was maintained for a period 2 h, the maximal time during which sperm motility could be maintained in vitro. Due to the dual role of NKAα4 in sperm motility and capacitation, ouabain derivatives allow for inhibition of sperm function not only while in the male reproductive tract but also in the female genital tract, where capacitation takes place.

As previously mentioned, the mechanisms of action by which NKAα4 supports sperm function are secondary to the maintenance of the Na^+^ and K^+^ ion gradients in the cells. Therefore, we tested whether compound **25** affected sperm plasma membrane potential, [Ca^2+^]*_i_*, and pH. Compound **25** at 10 nM caused sperm plasma membrane depolarization, increasing membrane potential by ~40%. It also reduced sperm cytosolic pH ~ 15% and increased [Ca^2+^]*_i_* ~40% [89]. These data show that compound **25** blocks vital parameters of sperm function, which all depend on NKAα4 activity. This agrees with the notion that compound **25** specifically targets NKAα4. However, different from ouabain, compound **25** exerts its effect at ~1000-fold lower amounts and in a NKAα4 isoform selective manner. Among other in vitro properties of compound **25** are its high metabolic stability, low toxicity in antiproliferative assays, and lack of interference with the human ether-a-go-go-related gene (hERG) K^+^ channel [89].

**Figure 10:**
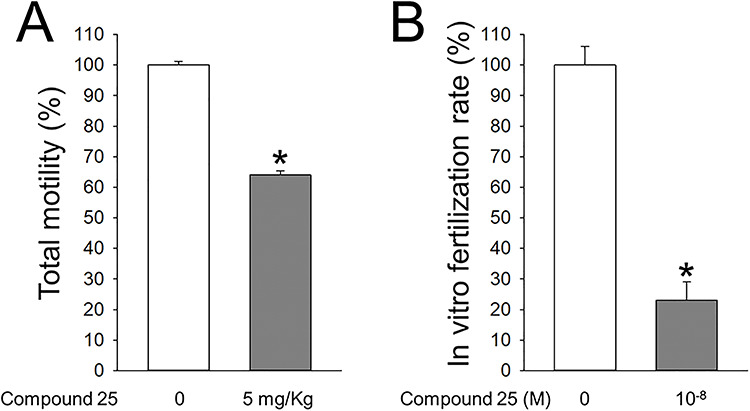
Effect of compound **25** on sperm motility after administration to rats and on in vitro fertilization. (A) Compound **25** was administered to rats by oral gavage at a dose of 5 mg/kg of body weight for 3 days. Then sperm motility was measured on sperm collected from the epididymis of the rats. (B) Effect of compound **25** on in vitro fertilization assays. Epididymal sperm was pretreated for 1 h with or without 10 nM of compound **25**. After placing sperm in contact with oocytes from hyper-ovulated rats, cultures were maintained for 24 h, and fertilization was estimated by the presence of the development of two cell embryos. Values are mean ± SEM.

Besides the in vitro effects of ouabain derivatives, compound **25** also had positive effects in vivo. Thus, when administered to rats daily via oral gavage at concentrations as low as 5 mg/kg, compound **25** inhibited total motility of sperm collected from the epididymis 3 days after initiation of the treatment (Figure 10A). These results show that compound **25** can not only interfere with sperm motility in vitro, but it also has activity after in vivo administration. In addition, compound **25** decreased the ability of the male gamete to fertilize oocytes in vitro by ~80% (Figure 10B). This represents a significant reduction when considering that in vitro fertilization assays highly maximize the chances of achieving egg fertilization.

Altogether, the in vitro and in vivo actions of compound **25** suggest that ouabain derivatives are attractive scaffolds to continue pursuing to achieve nonhormonal, reversible male contraception. We are currently performing mating trials to assess the effectiveness of ouabain derivatives as contraceptive agents.

## Final remarks

NKAα4 isoform is an example of the exquisite adaptation that nature has undergone to serve the unique mechanisms that allows sperm to swim. The transmembrane Na^+^ and K^+^ gradients generated by NKAα4 allow sperm to travel the long journey to reach the egg and to undergo changes in motility that are required for fertilization. Pharmacological targeting of such an essential system should provide a means to achieve male contraception. We have shown proof of principle for the use of the high ouabain sensitivity of NKAα4 as an approach to inhibit sperm’s active Na^+^ and K^+^ transport. Ouabain derivatives with modifications at the glycone (C3) and the lactone (C17) domains resulted in compounds, some of which have a high binding capacity and an outstanding selectivity profile for NKAα4. Compounds of this class affect sperm motility and hypermotility both in vitro and in vivo. This suggests that the compound is reaching the sperm following systemic dosing and that its action persists even after the cells are isolated from the rat epididymis. At this time, it is unknown if compound **25** has the capacity to cross the blood–testis barrier, or if it reaches sperm in the epididymis, or if it can even target the sperm later in the ejaculate, through its secretion via the different accessory glands present along the male reproductive tract. Additional experiments will be required to determine the distribution of ouabain analogs to different regions of the male reproductive tract. In addition, further experiments need to be carried out to establish the pharmacokinetic parameters and bioavailability of compound **25**. This will guide us in making potential chemical modifications that can enhance the properties of these compounds. Having additional chemical scaffolds with different physicochemical properties, metabolic stability, and pharmacokinetics will increase the possibilities for obtaining compounds with alternative advantageous characteristics. In conclusion, the synthetic cardenolides that we have generated provide original scaffolds with properties that make them appealing tools for the highly unmet goal of obtaining male contraception.
